# Vitamin D Deficiency, Chronic Kidney Disease and Periodontitis

**DOI:** 10.3390/medicina60030420

**Published:** 2024-02-29

**Authors:** Imaan Ganimusa, Emily Chew, Emily Ming-Chieh Lu

**Affiliations:** 1 Faculty of Dentistry, Oral and Craniofacial Sciences, King’s College London, London SE1 9RT, UK; 2 Centre for Host-Microbiome Interactions, Faculty of Dentistry, Oral and Craniofacial Sciences, King’s College London, London SE1 9RT, UK

**Keywords:** vitamin D, chronic kidney disease, periodontitis

## Abstract

Vitamin D has important anti-inflammatory, anti-microbial properties and plays a central role in the host immune response. Due to the crucial role of the kidneys in the metabolism of vitamin D, patients with chronic kidney disease (CKD) are prone to vitamin D deficiency. The resultant reduction in the production of calcitriol, the activated form of vitamin D, in patients with CKD is responsible for exacerbating the existing renal impairment and periodontal inflammation. Recent evidence suggests a bidirectional, causal relationship between periodontitis and renal functional status. Both conditions have shared pathophysiological mechanisms including oxidative stress, increases in the systemic inflammatory burden and impaired host response. This review explores the association between vitamin D, CKD and periodontitis. The review summarises the current evidence base for the classical and non-classical vitamin D metabolic pathways, the biological mechanisms linking vitamin D deficiency, CKD and periodontitis, as well as the bidirectional relationship between the two chronic inflammatory conditions. Finally, the paper explores the impact of vitamin D deficiency on CKD, periodontitis, and related co-morbidities.

## 1. Introduction

Vitamin D deficiency is associated with various noncommunicable diseases, among which are chronic kidney disease (CKD) and periodontal disease. As well as its fundamental roles in calcium and phosphate homeostasis, vitamin D possesses important antibacterial, anti-inflammatory and host modulatory effects [1], which exert “renoprotective” and “perio-protective” effects. 

CKD is a global health concern affecting 5–10% of the world population [2]. According to Kidney Disease Improving Global Outcomes (KDIGO), CKD is diagnosed on the basis of an estimated glomerular filtration rate (eGFR) and values less than 60 mL/min/1.73 m^2^ have been identified as the threshold for CKD [3,4], together with abnormalities of renal structure or function, present for more than 3 months with implications for health [4]. There are five stages in CKD, the higher the staging the lower the eGFR [4]. 

Periodontitis is a chronic multifactorial inflammatory disease with an overall prevalence of 11.2% and is the 6th most prevalent disease worldwide [5]. It is associated with dysbiosis of the oral flora, characterised by the progressive destruction of the periodontium, with loss of clinical attachment, loss of the alveolar bone, presence of periodontal pockets and gingival bleeding [6]. Periodontitis is a serious public health issue, as it can cause not only local symptoms, but it can also have a negative impact on the individual’s general health, contributing to the development, and to the worsening, of chronic non-communicable degenerative diseases, such as chronic kidney disease (CKD) [7].

Periodontal disease and CKD have shared pathophysiological mechanisms, namely an increased inflammatory state, impaired immune response, and oxidative stress [8]. Therefore, the occurrence of both conditions is likely to result in an amplification of adverse outcomes [9]. A recent large cohort study suggested a bidirectional, causal relationship between periodontal inflammation and renal function [10], such that a 10% increase in periodontal inflamed surface area (PISA) was associated with a 3.0% decrease in eGFR and a 10% decrease in eGFR led to a 25.0% increase in PISA [10].

This review will discuss (1) the vitamin D metabolic pathway and how this is altered in CKD patients; (2) the bidirectional relationship between periodontal inflammation and renal impairment; (3) the biological mechanisms linking vitamin D deficiency, CKD, and periodontitis; and (4) the impact of Vitamin D deficiency on systemic inflammation and co-morbidities associated with CKD and periodontitis. 

## 2. Vitamin D Metabolic Pathways

Vitamin D is a fat-soluble hormone that can be obtained from two main sources. Firstly, it can be obtained from dietary sources such as fatty fish and mushrooms. It is found in the form of Ergocalciferol (D2) from plant sources or Cholecalciferol (D3) from animal sources [11]. Secondly, it can be obtained through the action of sunlight’s ultraviolet rays on skin in the form of 7-Dehydrocholesterol which is converted into the previtamin Cholecalciferol (D3) [12]. Due to the relatively small proportion of vitamin D from the diet, dermal synthesis accounts for 90% of vitamin D provision [13]. 

### 2.1. Classical Pathway

**Figure 1:**
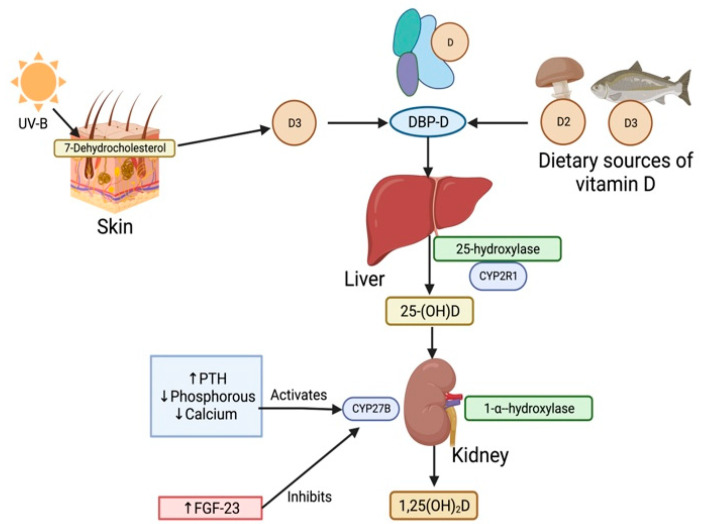
Vitamin ’s classical activation pathway in the human body. Sources of Vitamin D such as UV rays and diet deliver vitamin D in their precursor forms, D2 and D3 [11]. These precursors then bind to the Vitamin D-binding protein (DBP) at the site of synthesis and form the DBP-D protein complex. Vitamin D is then carried in the DBP-D complex through the blood plasma to the liver. Precursors D2 and D3 are hydroxylated into inactive 25-hydroxvitamin D [25(OH)D] by 25-hydroxylase, coded by the cytochrome P2R1 (CYP2R1) gene [14]. 25(OH)D is taken up into the kidney from the blood and activated via 1-α-hydroxylation by the CYP27B gene to the 1,25-dihydroxyvitamin D (1,25(OH)2D) activated state. An increase in parathyroid hormone (PTH), reductions in phosphorous and calcium upregulates CYP27B gene activity, while an increase in fibroblast growth factor (FGF-23) downregulates CYP27B gene activity [15].

The classical pathway for the activation of vitamin D involves two stages of hydroxylation. Firstly, the Vitamin D_2_ and D_3_ precursors are transported to the liver by vitamin D-binding protein (DBP) [14]. Precursors D2 and D3 are then converted into inactive 25-hydroxvitamin D (25(OH)D) by hydroxylation at the C25 position by 25-hydroxylase, coded by cytochrome P2R1 (CYP2R1) [15]. 25(OH)D acts as the main circulating and storage form of vitamin D in the body. 25(OH)D is then circulated in blood as the 25(OH)-DBP complex and undergoes glomerular filtration and uptake into the kidney proximal tubule cell by the receptor megalin. 25(OH)D-DBP then undergoes 1-α-hydroxylation by the Cytochrome P450 Family 27 Subfamily B Member 1 gene (CYP27B1) to its most activated state, 1,25-dihydroxyvitamin D (1,25(OH)2D), also known as calcitriol [15]. This is illustrated in Figure 1.

Calcitriol has wide ranging physiological and pharmacological effects [1]. Calcitriol is responsible for increasing intestinal calcium and phosphate absorption when serum calcium and phosphate levels are low. It also increases phosphorus resorption from bone and is involved in the production of antimicrobial peptides, epithelial defence mechanisms, host modulatory effects, the maintenance of the renin-angiotensin system, the inhibition of host tumour cells and suppression of parathyroid hormone (PTH) release [1]. Calcitriol exerts these affects by binding to intracellular vitamin D receptors (VDRs), which are steroid hormone nuclear receptors and function as transcription factors [16].

In health, the activation of the CYP27B gene is regulated by PTH, phosphorus, calcium, and fibroblast growth factor-23 (FGF-23) levels, and subsequently calcitriol levels [17]. Increased PTH levels combined with decreased phosphorus and calcium levels activate the CYP27B gene and lead to increased calcitriol levels [13]. Meanwhile, increased FGF-23 levels inhibit the CYP27B gene and decrease calcitriol levels [18].

### 2.2. Non-Classical Pathway

Aside from the classical metabolism of vitamin D and its role in calcium and phosphate homeostasis, a non-classical pathway of calcitriol synthesis appears to be present in various tissues, both including and peripheral to the kidneys. Additionally, 1-α-hydroxylase (which is primarily expressed in the kidneys) may also be expressed in extrarenal cells and tissues [19]. Thus, the extra-renal production of calcitriol primarily functions as an autocrine or paracrine factor at extra-renal sites and thus plays a role in the non-classical actions of vitamin D [20,21].

**Figure 2:**
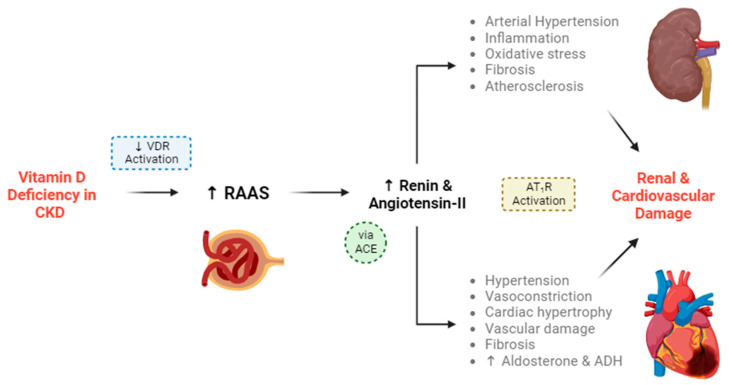
Vitamin D deficiency leads to the progression of chronic kidney disease (CKD) and cardiovascular disease (CVD). Vitamin D deficiency leads to a decrease in VDR-mediated functions. VDR activation is responsible for stimulation of the juxtaglomerular apparatus, which usually suppresses the renin–angiotensin–aldosterone system (RAAS) and the secretion of renin. In the absence of VDR activation (created by vitamin D deficiency), intracellular calcium is not stimulated within the juxtaglomerular apparatus, and RAAS activity increases. Renin and subsequent angiotensin-II production will increase in turn. Renin cleaves angiotensinogen into angiotensin I, which is then converted into angiotensin II by the angiotensin-converting enzyme (ACE). Angiotensin-II can produce more downstream angiotensin sub-types [23,24]. Angiotensin-II binds to the type 1 angiotensin-II receptor (AT_1_R) [25], increasing sympathetic tone [26], blood pressure [22,27], inflammation [28], fibrosis [29], aldosterone production [30], anti-diuretic hormone (ADH) production [31] and cardiac hypertrophy [32]. Aside from the vascular damage caused, AT_1_R activation increases vascular smooth muscle cell dedifferentiation, leading to atherosclerosis [33]. AT_1_R activation also decreases parasympathetic tone [34] and nitric oxide production [35], contributing to hypertension. These effects culminate as renal and cardiovascular damage [22,23,36]. Vitamin D works to protect against this damage by suppressing RAAS, but this suppression is reversed during CKD.

Central to the non-classical function is the regulation of the renin–angiotensin–aldosterone system (RAAS). Calcitriol is regarded as a negative endocrine regulator of the renin gene, thereby inhibiting the RAAS and preserving renal function. The RAAS stimulates the production of renin, which cleaves angiotensin into angiotensin I, which is then processed into angiotensin II by the angiotensin-converting enzyme (ACE). Angiotensin II binds to the type 1 angiotensin II receptor (AT_1_R) to produce various deleterious effects for renal and cardiovascular tissues, including hypertension [22,23]. This is described in more detail in Figure 2.

There is also evidence that VDR activation by calcitriol may also downregulate other RAAS components aside from renin, including the Ang II type one receptor, renin receptor and transforming growth factor beta [37]. This can aid in the reduction of renal blood pressure and fibrogenesis. VDR activation by calcitriol could also have anti-inflammatory effects, via the suppression of nuclear factor-kB (NF-kB) activation. It also appears that VDR can form complexes with various transcription factors and engage in crosstalk with a wide range of cellular signals [38], thus illustrating the depth of the relationship between VDR activation and RAAS components. 

The suppression of the NF-kB pathway by calcitriol has extra-renal consequences too. Its suppression of the pathway is twofold: calcitriol suppresses NF-kB nuclear migration and phosphorylation, and it downregulates IkB phosphorylation (a protein involved in NF-kB signalling) by suppressing ROS activity [39]. The NF-kB pathway promotes pro-inflammatory cytokine expression, and thus a reduction in NF-kB activity results in a reduction in inflammatory markers. Thus, the suppression of the NF-kB pathway in turn can prevent insulin resistance [40], have neuroprotective effects against ischaemic strokes [39], and protect against other inflammatory disorders. Therefore, both the inhibitions of the RAAS and the NF-kB pathway are responsible for the “reno-protective” effects of vitamin D [41].

It is also worth noting the role of vitamin D in muscle health, perhaps best characterised by the link between vitamin D deficiency and sarcopenia, a generalised degenerative skeletal muscle disorder [42,43]. Low serum 25(OH)D levels are associated with sarcopenia in haemodialysis patients [42]. This is explained by the fact that calcitriol binds to VDRs in skeletal myocytes, stimulating protein synthesis [44,45]. Patients suffering from renal failure have an increased risk for the development of sarcopenia, due to their accelerated protein catabolism, the dialysis procedure itself, as well as their low energy and protein intakes [46]. Therefore, replenishing vitamin D levels would facilitate the restoration of muscle health [46].

## 3. Serum 25(OH)D Thresholds

The serum 25-(OH)vitamin D level is the ideal indicator of deficiency. However, there is a lack of agreement in the defining serum concentrations associated with deficiency and adequacy [1]. Most guidelines currently define vitamin D sufficiency as any value above 50 nmol/L (20 ng/mL) [47,48]. However, various studies have indicated that optimal serum levels should be anywhere between 100 to 200 nmol/L (40–80 ng/mL) [49,50], and that a concentration below 50 nmol/L (20 ng/mL) is considered vitamin D deficient [51].

## 4. Biological Mechanisms Linking Vitamin D Deficiency, CKD and Periodontitis

Vitamin D plays a central role in the host immune response and possesses important anti-inflammatory, anti-microbial and host modulatory properties [1]. Due to the wide- ranging physiological and pharmacological role of calcitriol [1], vitamin D deficiency is responsible for the exacerbation of renal impairment and the progression of periodontitis. CKD is characterised by an altered vitamin metabolism, as well as elevations in PTH and FGF23. Additionally, the oxidative stress which promotes inflammation and an impaired host response provides the pathophysiological mechanisms for disease progression in both CKD and periodontitis. Finally, the vitamin D-binding protein (DBP) polymorphisms which are associated with bioavailable 25(OH)D are linked to the severity and progression of CKD and periodontitis.

### 4.1. Altered Vitamin D Pathways in CKD

The kidney plays a central role in the vitamin D metabolism and the regulation of its circulating levels. Vitamin D deficiency has been identified in more than 80% of patients with CKD [52]. The trend is that the deficiency worsens with progressive renal impairment, ultimately the onset of hyperparathyroidism. There are several mechanisms responsible for the reduced production of calcitriol: (1) in CKD, there is an overall reduction in renal mass, limiting the 1-α-hydroxylase available for the production of calcitriol [38]; (2) reduced eGFR also limits the conversion of 25(OH)D by 1-α-hydroxylase, further reducing the production of calcitriol [20]; (3) reduced renal megalin receptors in CKD will lead to the reduced uptake of 25(OH)D and therefore reduced production of calcitriol; (4) the elevation of FGF23 in CKD, inhibits the CYP27B gene [53,54], which also reduces 1-α-hydroxylase activity [55,56]; (5) hyperphosphatemia due to impaired renal phosphate excretion in CKD also contributes to reduced 1-α-hydroxylase activity [57]. Thus, the downregulation of 1-α-hydroxylase activity reduces the overall production of calcitriol from the kidneys.

While the inverse relationship between serum 25(OH)D and renal function has traditionally been explained by alterations in the classical vitamin D metabolism (Section 2.1), it is clear that non-classical functions of calcitriol also play a role (Section 2.2). A deficiency in vitamin D and thus a reduction in calcitriol promotes RAAS activity, and the sequential activation of angiotensin II could raise blood pressure and damage the renal microvasculature. Aside from its direct action on the RAAS, vitamin D deficiency promotes insulin resistance via a wide range of molecular mechanisms involved in glucose homeostasis and immune modulation [58], which could lead to diabetic nephropathy and subsequent RAAS activation [59]. 

Indeed, intrarenal angiotensin II levels may be up to a hundred times higher in diabetic patients [59], and diabetic nephropathy remains a leading cause of CKD [60]. Therefore, RAAS activation is an important factor in the progression on CKD, and a deficiency in vitamin D both directly and indirectly promotes RAAS activity. However, it should be noted that there is a lack of evidence linking vitamin D supplementation to the slowed progression of CKD. 

Locally synthesised angiotensin II is also detrimental to the cardiovascular system, and cardiovascular disease is responsible for much of the mortality of CKD [61]. In these ways, a deficiency in vitamin D can lead to profound consequences for renal, endocrine and cardiovascular function, all of which exacerbate the development and severity of CKD.

### 4.2. Elevations in PTH and FGF23 Levels in CKD

Vitamin D levels are also regulated by PTH and FGF23. Increases in the serum levels of FGF23 and PTH are responsible for vitamin D deficiency (Figure 1). PTH increases renal calcium reabsorption, the excretion of phosphorus, and stimulates calcitriol synthesis [62,63].

FGF23 is a phosphaturic hormone secreted by osteoblasts and osteocytes which is strongly associated with inflammation. The elevation of FGF23, which is to offset phosphorus retention in CKD, inhibits the renal expression of 1-a-hydroxylase [64], and reduces the production of calcitriol. Thus, serum FGF23 increases with the decline in eGFR and an increased phosphate level. This results in the downstream reduction of 1,25(OH)D concentrations and the onset of secondary hyperparathyroidism (SHPT) [64,65] due to elevated PTH but low or normal calcium levels. SHPT ultimately progresses to tertiary hyperparathyroidism (THPT), where both the PTH and calcium levels are elevated [66]. As a result, vitamin D deficiency (<20 ng/mL) and insufficiency (20–29 ng/mL) are common in individuals with CKD [67].

### 4.3. Oxidative Stress

Oxidative stress is a consequence of vitamin D deficiency and plays a role in the disease progression of both CKD and periodontitis [8]. The detrimental impact of oxidative stress on the body derives from its promotion of inflammation by stimulating the release of pro-inflammatory medicators (via the NF-kB-related cascade) [68]. Vitamin D downregulates various intracellular oxidative stress-related pathways, including the enhanced expression of the nuclear factor, erythroid-2(Nf-E2)-related factor (Nrf2) and Klotho [69,70,71]. Klotho is a family of proteins involved in antioxidant production [72], expressed mainly in the kidneys [73], but also in the human alveolar bone [74]. The α-Klotho protein promotes FGF23 signalling in both renal and periodontal tissues, which regulates vitamin D homeostasis [75,76]. In renal tissues, α-Klotho has been shown to reduce oxidative stress by modulating NF-kB expression [77], and preventing other delirious effects of CKD [75]. In periodontal tissues, α-Klotho has been shown to resist the oxidative stress-related apoptosis of periodontal ligament stem cells, and maintain the antioxidant capacity in these cells [78]. In fact, α-Klotho serum levels have been shown to be indirectly proportional to periodontitis severity [76]. In these ways, vitamin D deficiency can lead to oxidative stress, namely via the subsequent reduction in Klotho expression. 

The progressions of CKD and periodontitis are both linked to oxidative stress, and so the progression of one morbidity can encourage the progression of the other. A recent animal study showed that renal tissue damage is linked to oxidative stress following periodontitis [79]. This concept was reinforced in a recent longitudinal study that pointed to oxidative stress, rather than inflammatory load, as the biological basis for the bidirectional relationship between impaired renal function and periodontitis [10]. Thus, oxidative stress is responsible for progressive renal impairment [80], tissue damage in periodontitis [80], as well as systemic implications for the development of atherosclerosis [81], and cardiovascular disease [82]. Interestingly, oxidative stress is considered a non-traditional risk factor for all-cause mortality [83,84].

### 4.4. Impaired Host Response

**Figure 3:**
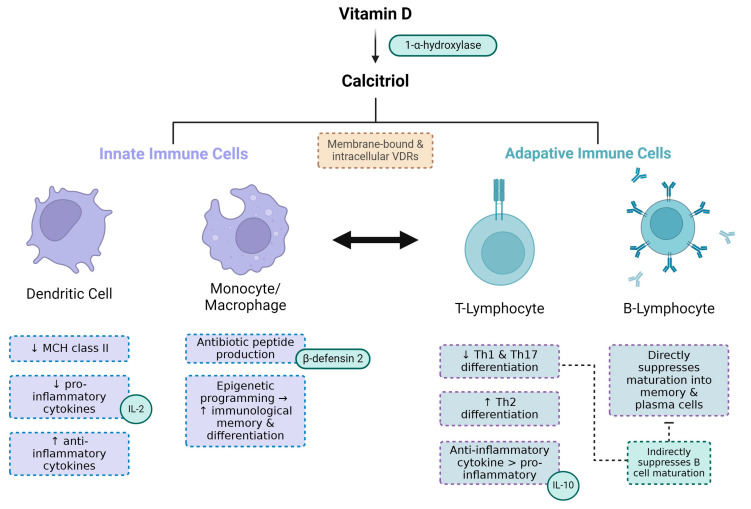
Vitamin D modulates the functions of various immune cells. 25-hydroxyvitamin D (vitamin D) is metabolised into its active form 1,25-dihydroxyvitamin D (calcitriol) by CYP27BQ (1-α-hydroxylase) [1]. Calcitriol binds to vitamin D receptors (VDRs) both on the cell-surface (membrane-bound VDRs) and in the cytoplasm (intracellular VDRs) of immune cells [87]. Calcitriol downregulates the production of MHC class II in dendritic cells, which is necessary for antigen recognition and dendritic cell activation. Activated dendritic cells stimulate T-lymphocyte (T-cell) activity, so the suppression of dendritic cells leads to reduced T-cell function [88,89]. The influence of innate dendritic cells in the adaptive T-cell response demonstrates the crosstalk between innate and adaptive immunity (as highlighted by the arrow). Calcitriol also suppresses the production of pro-inflammatory cytokines like interleukin-2 (IL-12) in dendritic cells, whilst stimulating the production of anti-inflammatory cytokines. Calcitriol binds to intracellular VDRs in macrophages and their monocyte precursors, forming heterodimers with the retinoid-X receptor [33]. This stimulates the production of cell membrane-destroying antibiotic peptides, including β-defensins [70,90]. Calcitriol also epigenetically regulates the immunological memory and differentiation of macrophages and monocytes [91]. Calcitriol reduces the differentiation of T-helper cells into types Th1 and Th17 and their production of pro-inflammatory cytokines [92,93], whilst stimulating their differentiation into the Th2-type and favouring their production of anti-inflammatory cytokines like interleukin 10 (IL-10). T-cell-derived pro-inflammatory cytokines are important for B-lymphocyte (B-cell) differentiation, and so the suppression of T-cell pro-inflammatory activity also suppresses B-cell activity. Calcitriol also directly suppresses naïve B-cell differentiation and maturation into memory and plasma cells [94].

The mechanisms linking vitamin D status, CKD and periodontitis are related to the biological functions of calcitriol, which possesses various immunomodulatory properties that affect both the innate and adaptive immune system. Calcitriol downregulates the expression of MHC class II (and co-stimulatory molecules) on antigen-presenting dendritic cells (Figure 3). It also suppresses pro-cytokine production—whilst stimulating anti-inflammatory cytokine production—and both these mechanisms suppress subsequent T-cell activation and differentiation [85,86]. 

Vitamin D exercises various effects on macrophages and their monocyte precursors, which are involved in phagocytosis and the cytokine (Figure 3). Upon pathogen recognition and antigen presentation, monocytes and macrophages upregulate VDR expression and metabolise vitamin D into calcitriol. Calcitriol activates intracellular VDRs to promote the production of antibiotic peptides including β-defensins and cathelicidins [90,95,96]. Calcitriol also epigenetically regulates the immunological memory and differentiation of monocytes and macrophages (Figure 3) [97].

Calcitriol also suppresses the activity of T- and B-lymphocytes. It reduces the differentiation of T-helper cells into the Th1-type and pro-inflammatory cytokine generation, in favour of Th2-type differentiation and the production of anti-inflammatory cytokines [92]. It also suppresses Th17-type differentiation and its inflammatory cytokines [93]. Calcitriol also suppresses naïve B-cell differentiation and their maturation into memory and plasma cells [94].

Overall, a lack of calcitriol promotes chronic inflammation through the inhibition of the aforementioned mechanistic pathways. Therefore, a vitamin D deficiency enhances the risk of both CKD and periodontitis through an impaired host response. 

### 4.5. DBP Genetic Polymorphisms and Bioavailable 25(OH)D

DBP serves to transport vitamin D and its metabolites such as 25(OH)D in the blood to specific target tissues where vitamin D will exert its biological effects. Only the bioavailable 25(OH)D, rather than the total serum 25(OH)D, is associated with serum calcium, and plasma PTH concentrations in patients on haemodialysis [98]. Bioavailable forms of vitamin D include the free fraction (<1% of total 25(OH)D) and the fractions bound to albumin or lipoprotein which are 10–15% of the total 25(OH)D. 

DBP genetic polymorphism rs7041 and rs4588 result in different phenotypes of the DBP that have varying binding affinities to 25(OH)D [10]. These polymorphisms have been associated with differences in bioavailable levels of vitamin D and have been implicated in the elevated risk of CKD [99] as well as periodontitis [100].

Additionally, DBP polymorphisms potentially explain the racial predilection for a particular DBP phenotype [101,102], as well as the differential responses to vitamin D supplementation in patients with low serum total 25(OH)D concentrations [103]. Thus, DBP polymorphisms can impact the relationship between serum total 25(OH)D concentrations and clinical outcomes and the effect of vitamin D supplementation [104]. 

Therefore, it may be inappropriate to assess the vitamin D status of individual patients using serum total 25(OH)D concentrations alone. Rather, the DBP phenotype (affinity) should be taken into account, due to the likely implications in the clinical prognosis of CKD and periodontitis, as well as responses to vitamin D supplementation. 

## 5. Impact of CKD on Periodontal Inflammation

Longitudinal studies have demonstrated an association between CKD and the progression of periodontal disease [105]. CKD is associated with a two-fold increase in the prevalence of periodontitis [9]. A recent meta-analysis also suggested that individuals with CKD presents with higher mean PPD, and CAL, compared to healthy subjects without CKD. The difference in PPD and CAL between CKD and healthy subjects was 0.25 mm and 0.041 mm, respectively [106]. 

### CKD- Mineral and Bone Disorder and Impact on Periodontitis

Homeostatic imbalances associated with CKD lead to changes in the regulation of calcium, phosphorous, PTH, fibroblast growth factor 23 (FGF23) and sclerostin levels, resulting in increased bone demineralisation, often referred to as CKD–mineral and bone disorder (CKD-MBD) [107]. In failing kidneys, there is a significant reduction in the hydroxylation of inactive vitamin D (25-hydroxvitamin D) [25(OH)D] into active calcitriol (1,25(OH)2D) by the 1-α-hydroxylase enzyme [1]. Hence, reduced calcitriol levels combined with reduced renal phosphate excretion lead to systemic hypocalcaemia and hyperphosphataemia, and secondary hyperparathyroidism. Eventually, secondary hyperparathyroidism progresses to tertiary hyperparathyroidism and the development of hyperparathyroid bone disease that is characterised by high bone turnover, a thinned cortical bone, and an increased abnormal trabecular bone [107]. 

CKD-MBD can exacerbate periodontitis by accelerating alveolar bone loss [108]. This concept was reinforced in an animal study where mice with chronic uraemia and hyperparathyroidism showed a significantly reduced cortical alveolar bone compared to healthy controls, and that a further decrease in bone levels was seen after a high phosphate diet was given to increase PTH hormone serum levels [109]. Furthermore, studies suggest that elevated levels of sclerostin in CKD increase the progression of periodontitis by inhibiting bone remodelling [110,111,112] and therefore, elevated sclerostin levels secondary to CKD-MBD may potentiate periodontitis-related alveolar bone loss [112,113]. 

## 6. Impact of Periodontal Inflammation on Renal Function

Large epidemiologic surveys such the NHANES III [114] have demonstrated that periodontitis has been predictive for the occurrence of CKD [79,87,115]. In particular, periodontitis has been identified as a non-traditional risk factor for eGFR decline [116], and a contributor of oxidative stress [10]. Furthermore, *P. gingivalis* lipopolysaccharide (LPS) exposure resulted in the elevation of FGF23 in the kidneys [117]. FGF23 has been identified as a risk factor for cardiovascular mortality in CKD patients [67,118,119]. Another possible link between decreased renal function and periodontal inflammation is the decreased levels of Fetuin-A [120]. Fetuin A is downregulated by common pro-inflammatory mediators associated with periodontitis such as: tumour necrosis factor alpha (TNFα), IL-1, and IL-6 [121]. As the severity of periodontitis progresses, Fetuin A levels decrease [122]. Low Fetuin A levels are linked to overall declining renal function, and increased renal calcification, but also notably an increased risk of endothelial dysfunction and cardiovascular mortality rates in CKD patients [123,124,125]. 

Recently, it has been shown that the presence of periopathogenic bacteria resulted in an elevation of TNFα that was predictive of the severity of renal impairment reflected by eGFR and periodontal clinical parameters such as the plaque index (PI), gingival index (GI), probing pocket depths (PPD) and clinical attachment loss (CAL) [126]. A meta-analysis suggested that periodontitis significantly increased the risk of all-cause mortality in CKD [127]. However, this result was refuted by a large database study from Taiwan—where it was concluded that periodontitis was not a predictor for long-term mortality or morbidity in patients with advanced CKD [128]. Therefore, future well-designed prospective studies are needed to verify these findings. 

Further evidence supporting the impact of periodontal inflammation on renal function comes from improved renal outcomes following non-surgical periodontal treatment. Recent systematic reviews and meta-analyses which have only included a limited number of studies suggest that periodontal treatment improved renal function in CKD patients [129,130]. This is also demonstrated by two case series studies, which showed improved eGFR and creatinine, as well as periodontal clinical outcomes at 3–6 months after non-surgical periodontal therapy [131]. The biological plausibility underlying the favourable outcomes relates to the shared pathophysiologic mechanisms between CKD and periodontitis including oxidative stress and an impaired host response (Section 4). Therefore, these findings reinforce the impact of periodontal inflammation on the progression of renal failure.

## 7. Impact of Vitamin D Deficiency on CKD

Vitamin D deficiency is associated with a higher risk of mortality, secondary and tertiary hyperparathyroidism [132]. Due to impaired renal function, the eGFR is reduced, and there is therefore a decline in the conversion of 25(OH)D to calcitriol, the active form of vitamin D. The latter reduces intestinal calcium absorption and, together with phosphate retention, contributes to the onset of secondary and tertiary hyperparathyroidism. 

CKD Patients who are vitamin D deficient have high mortality rates [133] and an increased cardiovascular risk [134]. In addition, the elevation of FGF23, which is linked with vitamin D deficiency, is associated with the progression of CKD towards end-stage renal disease (ESRD), the occurrence of cardiovascular (CVS) events and increased mortality rates in patients with CKD [118,119]. 

The serum total 25(OH)D concentrations were predictive of renal outcomes, such as the doubling of serum creatinine in ESRD, and associated with disease progression and morality [55,135]. A meta-analysis of prospective studies demonstrated an increased relationship between all-cause mortality in patients with CKD and serum total 25(OH)D concentrations [136]. Conversely, a recent systematic review and meta-analysis concluded that higher levels of serum 25(OH)D were associated with lower risks of all-cause mortality [137].

Therefore, to ensure that patients with CKD avoid vitamin D deficiency and prevent complications such as secondary and tertiary hyperparathyroidism and other co-morbidities [67,138,139], the Kidney Disease Outcomes Quality Initiative (KDOQI) and Kidney Disease Improving Global Outcomes (KDIGO) group have suggested the use of vitamin D supplementation [138,139].

### Vitamin D Supplementation in CKD Patients

Vitamin D supplementation is associated with the reduced risk of all-cause mortality [140] and cardiovascular mortality in patients in CKD, including those with ESRD [92,139]. In particular, therapies with calcitriol and analogues are associated with reduced mortality in CKD patients, particularly those suffering from SHPT [141].

CKD patients are deficient in vitamin D, even in the early stages of the disease [61]. Vitamin D plays a vital role not only in mineral homeostasis, but also in systemic health. As such, it is advised that vitamin D supplementation in CKD patients begins as soon as possible, to ensure a pool of vitamin D can be turned into calcitriol. 

During the early stages of CKD, where there is still evidence of residual renal function, supplementation can be achieved for CKD patients with oral forms of inactive vitamin D_3_ or D_2_. Vitamin D_3_ (cholecalciferol) is the natural form synthesised in the dermis, whilst vitamin D_2_ (ergocalciferol) is a synthetic product made using fungi [142]. Another reason is that vitamin D_2_ is associated with higher catabolic processes, and therefore, the overall improvement in serum vitamin D levels is not as sustainable as that seen with D_3_. Therefore, vitamin D_3_ is superior to vitamin D_2_ in raising the total 25(OH)D and thus ideally used for supplementation [143]. 

It is important to appreciate that the renal production of calcitriol becomes suppressed during Stages 3–4 of CKD [49,50] that are characterised by the significant loss of renal 1-α-hydroxylase, and the development of SHPT with declining kidney function. The current KDIGO guidelines recommend that calcitriol and vitamin D analogue supplementation should be reserved for predialysis CKD patients with severe and progressive hyperparathyroidism [2]. However, in line with the above points and existing clinical research [49,50,144], vitamin D supplementation (ideally with D_3_) should begin immediately after diagnosis to facilitate endogenous conversion into calcitriol and its positive downstream effects. 

## 8. Impact of Vitamin D Deficiency on Periodontitis

The relationship between vitamin D and periodontitis was recently reviewed [1]. In general, an inverse association exists between serum 25(OH)D and periodontal disease inflammation. Most of the studies supporting this association were cross-sectional or case–control studies. One of these was the NHANES III study which showed that low serum vitamin D was associated with periodontal inflammation [145]. 

Vitamin D supplementation may improve clinical outcomes in patients with periodontitis. One RCT demonstrated that the administration of vitamin D (700 IU/day) and calcium (500 mg/day) significantly reduced tooth loss in older patients during three years of observation [146]. While some studies have demonstrated moderately improved short-term periodontal outcomes after non-surgical periodontal therapy in patients on vitamin D supplementation [147,148,149], others have not [150]. One RCT demonstrated improved clinical and radiographic outcomes following periodontal surgery in patients where presurgical teriparatide, which is a commercially available PTH, was provided [151,152]. However, a 2020 meta-analysis concluded further investigations are needed before robust conclusions could be drawn on the benefits of vitamin D supplementation in periodontitis patients [153].

Interestingly, it was shown in a case–control study that patients with CKD and periodontitis showed lower serum levels of vitamin D compared to control patients without periodontitis [154]. In other words, vitamin D deficiency was more severe in patients with CKD and periodontitis than patients with CKD only [151]. This is plausible, given the shared pathophysiologic mechanisms between CKD and periodontitis, including the elevation of pro-inflammatory cytokines, impaired host response cytokines, and the increase in oxidative stress.

### Impact of Vitamin D Deficiency on Periodontitis in Pregnant Women and Adverse Pregnancy Outcomes

The impacts of pregnancy hormone and immunological changes on the increased risk of periodontal inflammation is well documented [155]. Periodontitis may potentiate adverse pregnancy outcomes, including preterm birth (PTB), and low birth weight (LBW) [155,156], via the direct actions of periopathogens on the feto-placental unit [157,158] and/or the increased levels of periodontitis associated proinflammatory cytokines that potentiate placental inflammation [159]. A deficiency in vitamin D further elevates the systemic inflammatory burden, adversely affecting the feto-placental unit and periodontal health of pregnant women. A recent study demonstrated a correlation between vitamin D deficiency, periodontitis and the prevalence of PTB and LBW in pregnant women [160]. Other recent studies have also demonstrated an association between vitamin D deficiency and an increased risk of PTB [161,162], and therefore it was suggested that pregnant women should receive vitamin D supplementation where necessary, to reduce the risk of PTB [162]. 

## 9. Impact of Vitamin D Deficiency on Co-Morbidities Associated with CKD and Periodontitis

Vitamin D has wide-ranging roles in promoting inflammation and reducing the risk of various co-morbidities. Therefore, aside from exacerbating CKD and periodontitis, a deficiency in vitamin D can also negatively and independently affect systemic health. The impact of vitamin D deficiency on systemic health will be specifically discussed here for cardiovascular disease, diabetes mellitus, autoimmune disease. However, as both CKD and periodontitis are directly and independently associated with a myriad of co-morbidities [83,163,164,165], it can be challenging to establish whether it is the contribution from vitamin D deficiency or the cause and effect of CKD and periodontitis that is driving the systemic inflammatory burden.

### 9.1. Cardiovascular Disease

A severe deficiency in vitamin D is positively correlated with a higher risk of cardiovascular disease (CVD) [166]. This is partly due to the reduction of cardiovascular risk factors by vitamin D, but also due to the direct effects of vitamin D on vascular tissues and cardiomyocytes, which express VDRs that respond to calcitriol [167]. Calcitriol has various positive effects on the vascular wall, namely reduced thrombogenicity and vasoconstriction, the inhibition of atherogenesis and the promotion of endothelial repair [168]. These effects protect against atherosclerosis and hypertensive damage. 

In cardiomyocytes, calcitriol regulates the intracellular calcium metabolism [169]. Calcitriol binds to VDRs on the cell-surface and in the cytoplasm. Membrane-bound receptors activate adenylate cyclase, which increases cytoplasmic calcium via downstream pathways. Cytosolic receptors, complex with retinoid-X receptors, migrate to the nucleus and upregulate the synthesis of the calcium-binding protein cholecalcin. 

Interestingly, the cardiovascular synthesis of calcitriol from its vitamin D precursor can be regulated by PTH, as is known to be the case in renal tissues [170]. Calcitriol is a known inhibitor of PTH action [171]. In this way, a deficiency in serum vitamin D (and therefore calcitriol) can lead to secondary and tertiary hyperparathyroidism, which has its own consequences for cardiovascular health. These include an increase in oxidative stress, the RAAS, thrombogenicity and foam cell formation, which can all lead to cardiovascular disease [167]. Given the fact that nutritional rickets, hypocalcaemia and SHPT have all been associated with heart failure [172], this PTH-mediated pathway could explain the negative cardiovascular effects of vitamin D deficiency, aside from VDR activation. 

CVD and its associated mortality are significant concerns for patients with CKD [173]. This is particularly true for CKD-MBD patients, and the presence of vascular calcifications provides an important assessment of cardiovascular risk for these patients, as per the KDIGO [2]. The presence of vascular calcifications is assessed based on the plain x-ray-based Adragao score, that is a good predictor of all-cause and CVD mortality in CKD-MBD patients [174], emphasising the link between CKD and CVD. 

Studies have also shown that periodontitis is also a major contributor to the development of atherosclerosis by potentiating endothelial dysfunction, inflammation, and the advancement of atherosclerotic plaque [164]. As such, periodontitis is considered to be independently linked to cardiovascular morbidity in patients with CKD [165].

### 9.2. Diabetes Mellitus

Diabetes mellitus is largely characterised by insulin resistance, where systemic inflammation plays a key role [175]. Diabetic nephropathy is responsible for almost 50% of ESRD cases [176]. the serum 25(OH)D levels of patients with type II diabetic nephropathy have been linked to renal disease progression [177]. In patients with diabetes, periodontitis is also independently associated with the progression of renal disease [178], while individuals suffering with diabetic nephropathy have a higher risk of periodontitis resulting in missing teeth compared to those patients without CKD [179]. 

Calcitriol protects tissues against inflammatory damage, by suppressing the systemic production of pro-inflammatory cytokines whilst encouraging the release of anti-inflammatory cytokines [180], as well as playing a protective role against insulin resistance by modulating pancreatic β cell activity [181]. Therefore, vitamin D deficiency could lead to insulin resistance and potentiate the development of diabetes mellitus. 

Calcitriol also maintains insulin secretion by β-cells, which, when reduced, can lead to the development of DM. It modulates calcium-mediated exocytosis, which is necessary for the secretion of insulin vesicles [182]. During the progression of insulin resistance, the β-cells secrete more insulin in response and this hyperactivity results in β-cell dysfunction and eventually apoptosis [183]. Calcitriol also controls intracellular reactive oxygen species (ROS) levels, by promoting the expression of cellular antioxidants, maintaining mitochondrial function [184], maintaining redox homeostasis [185] and decreasing nitrogen oxide production [186]. Vitamin D also regulates the target cell response to insulin by promoting insulin receptor expression [187]. Vitamin D deficiency can lead to secondary and tertiary hyperparathyroidism, which are also linked to glucose intolerance and insulin resistance [188]. In these ways, calcitriol protects the tissues against insulin resistance. 

Vitamin D also influences DM epigenetically. It suppresses the hypermethylation of diabetes-related genes by increasing the expression of demethylases, ensuring that those genes remain inactivated [189,190]. 

### 9.3. Autoimmune Disease

Vitamin D deficiency is correlated with the development of various autoimmune diseases [191]. Rheumatoid arthritis (RA) is an autoimmune disease of the joints, characterised by chronic synovial inflammation. Various studies have found a negative correlation between serum vitamin D levels and disease severity [191,192], though this could be blamed—at least in part—on the associated lack of mobility and sunlight exposure in RA patients [193]. Even so, a decrease in vitamin D is associated with the increased TNF-α and interleukin-6 (IL-6) secretion by inflammatory cells, and the suppression of endothelial function [194]. The pharmacological use of calcitriol in RA patients has been shown to inhibit pro-inflammatory cytokines and matrix metallopeptidase production in synoviocytes [195], reducing the recruitment of inflammatory cells to the site and consequent joint destruction. In these ways, vitamin D deficiency could contribute to the onset or progression of rheumatoid arthritis. 

Systemic lupus erythematosus (SLE) is an autoimmune disease characterised by systemic inflammation and the presence of immune complexes. SLE patients tend to have lower serum vitamin D levels, and there is some evidence of a relationship between vitamin D levels and disease severity [195,196,197]. The main reason for this association is due to the role of calcitriol, which suppresses T lymphocytes and the antinuclear antibody production by B lymphocytes and thus dampens the formation of immune complexes [198]. However, the depth of the relationship between SLE and vitamin D is still poorly understood. 

Multiple sclerosis (MS) is an autoimmune disease of the central nervous system and is more common in high-latitude regions where sunlight exposure and consequent vitamin D synthesis are limited [199]. It is known that reduced serum vitamin D levels are negatively correlated with disease severity in MS [200]. Calcitriol inhibits the differentiation and proliferation of type 1 T helper (Th1) cells by promoting the production of pro-inflammatory cytokines, such as IL-10 and transforming growth factor beta (TGF-β), over anti-inflammatory cytokines (such as IL-12 and TNF-α) in dendritic cells [86,201,202]. The anti-inflammatory effects of calcitriol on dendritic cells in the CNS can suppress the onset or exacerbation of MS, thus increasing the risk of MS without vitamin D and calcitriol. 

Vitamin D deficiency can also be implicated with various autoimmune disorders of the endocrine system, including diabetes mellitus, Hashimoto’s thyroiditis, and Addison’s disease. One possible mechanism is the production of autoantibodies associated with VDR polymorphisms [203,204,205]. 

## 10. Conclusions

A wealth of evidence supports the association between Vitamin D deficiency and chronic inflammatory conditions such as chronic kidney disease and periodontitis. Both conditions share common pathophysiologic mechanisms including insufficient inflammation, an impaired host response and oxidative stress. The kidneys play a crucial role in the metabolism of vitamin D. Thus, altered vitamin D metabolic pathways and elevations of PTH and FGF23 are key biochemical observations in CKD. Emerging evidence also supports the bidirectional relationship between renal impairment and periodontal inflammation. Vitamin D plays a significant role in the host immune response and possesses important anti-inflammatory, anti-microbial and host modulatory properties. Vitamin D status is also associated with renal outcomes and clinical outcomes related to periodontitis. However, as many of these studies were based on large observational studies, further prospective randomised controlled trials are needed to provide deeper insights into this relationship.

## Data Availability

Not applicable.

## References

[1] Lu E.M.C. (2023). The role of vitamin D in periodontal health and disease. J. Periodontal Res..

[2] Kidney Disease: Improving Global Outcomes (KDIGO) CKD-MBD Update Work Group (2017). KDIGO 2017 Clinical Practice Guideline Update for the Diagnosis, Evaluation, Prevention, and Treatment of Chronic Kidney Disease-Mineral and Bone Disorder (CKD-MBD). Kidney Int. Suppl..

[3] Schaeffner E.S., Ebert N., Delanaye P., Frei U., Gaedeke J., Jakob O., Kuhlmann M.K., Schuchardt M., Tölle M., Ziebig R. (2012). Two novel equations to estimate kidney function in persons aged 70 years or older. Ann. Intern. Med..

[4] Stevens P.E., Levin A. (2013). Kidney Disease: Improving Global Outcomes Chronic Kidney Disease Guideline Development Work Group Members. Evaluation and management of chronic kidney disease: Synopsis of the kidney disease: Improving global outcomes 2012 clinical practice guideline. Ann. Intern. Med..

[5] Tonetti M.S., Jepsen S., Jin L., Otomo-Corgel J. (2017). Impact of the global burden of periodontal diseases on health, nutrition and wellbeing of mankind: A call for global action. J. Clin. Periodontol..

[6] Papapanou P.N., Sanz M., Buduneli N., Dietrich T., Feres M., Fine D.H., Flemmig T.F., Garcia R., Giannobile W.V., Graziani F. (2018). Periodontitis: Consensus report of workgroup 2 of the 2017 World Workshop on the Classification of Periodontal and Peri-Implant Diseases and Conditions. J. Periodontol..

[7] Parsegian K., Randall D., Curtis M., Ioannidou E. (2022). Association between periodontitis and chronic kidney disease. Periodontol. 2000.

[8] Tonetti M.S., Van Dyke T.E., on behalf of Working Group 1 of the Joint EFP/AAP Workshop (2013). Periodontitis and atherosclerotic cardiovascular disease: Consensus report of the Joint EFP/AAP Workshop on Periodontitis and Systemic Diseases. J. Periodontol..

[9] Deschamps-Lenhardt S., Martin-Cabezas R., Hannedouche T., Huck O. (2019). Association between periodontitis and chronic kidney disease: Systematic review and meta-analysis. Oral Dis..

[10] Sharma P., Fenton A., Dias I.H.K., Heaton B., Brown C.L., Sidhu A., Rahman M., Griffiths H.R., Cockwell P., Ferro C.J. (2021). Oxidative stress links periodontal inflammation and renal function. J. Clin. Periodontol..

[11] Benedik E. (2022). Sources of vitamin D for humans. Int. J. Vitam. Nutr. Res..

[12] Lehmann B., Genehr T., Knuschke P., Pietzsch J., Meurer M. (2001). UVB-Induced Conversion of 7-Dehydrocholesterol to 1α,25-Dihydroxyvitamin D3 in an In Vitro Human Skin Equivalent Model. J. Investig. Dermatol..

[13] Chang S.W., Lee H.C. (2019). Vitamin D and health—The missing vitamin in humans. Pediatr. Neonatol..

[14] Bikle D.D. (2014). Vitamin D metabolism, mechanism of action, and clinical applications. Chem. Biol..

[15] Christakos S., Ajibade D.V., Dhawan P., Fechner A.J., Mady L.J. (2010). Vitamin D: Metabolism. Endocrinol. Metab. Clin. N. Am..

[16] Rochel N., Wurtz J.M., Mitschler A., Klaholz B., Moras D. (2000). The crystal structure of the nuclear receptor for vitamin D bound to its natural ligand. Mol. Cell..

[17] Silver J., Naveh-Many T. (2013). FGF-23 and secondary hyperparathyroidism in chronic kidney disease. Nat. Rev. Nephrol..

[18] Nakashima A., Yokoyama K., Yokoo T., Urashima M. (2016). Akio Nakashima, Keitaro Yokoyama, Takashi Yokoo, Mitsuyoshi Urashima. World J. Diabetes.

[19] Hewison M. (2010). Vitamin D and the immune system: New perspectives on an old theme. Endocrinol. Metab. Clin. N. Am..

[20] Dusso A.S. (2011). Kidney disease and vitamin D levels: 25-hydroxyvitamin D, 1,25-dihydroxyvitamin D, and VDR activation. Kidney Int. Suppl..

[21] Adams J.S., Hewison M. (2010). Update in Vitamin D. J. Clin. Endocrinol. Metab..

[22] Maranduca M.A., Clim A., Pinzariu A.C., Statescu C., Sascau R.A., Tanase D.M., Serban D.N., Branisteanu D.C., Branisteanu D.E., Huzum B. (2023). Role of arterial hypertension and angiotensin II in chronic kidney disease (Review). Exp. Ther. Med..

[23] Paz Ocaranza M., Riquelme J.A., García L., Jalil J.E., Chiong M., Santos R.A., Lavandero S. (2020). Counter-regulatory renin-angiotensin system in cardiovascular disease. Nat. Rev. Cardiol..

[24] Ferrario C.M. (2006). Role of angiotensin II in cardiovascular disease—Therapeutic implications of more than a century of research. JRAAS J. Renin-Angiotensin-Aldosterone Syst..

[25] Forrester S.J., Booz G.W., Sigmund C.D., Coffman T.M., Kawai T., Rizzo V., Scalia R., Eguchi S. (2018). Angiotensin II signal transduction: An update on mechanisms of physiology and pathophysiology. Physiol. Rev..

[26] Huang B.S., Chen A., Ahmad M., Wang H.W., Leenen F.H.H. (2014). Mineralocorticoid and AT1 receptors in the paraventricular nucleus contribute to sympathetic hyperactivity and cardiac dysfunction in rats post myocardial infarct. J. Physiol..

[27] Iyer S.N., Lu D., Katovich M.J., Raizada M.K. (1996). Chronic control of high blood pressure in the spontaneously hypertensive rat by delivery of angiotensin type 1 receptor antisense. Proc. Natl. Acad. Sci. USA.

[28] Wolf G., Wenzel U., Burns K.D., Harris R.C., Stahl R.A.K., Thaiss F. (2002). Angiotensin II activates nuclear transcription factor-κB through AT1 and AT2 receptors. Kidney Int..

[29] Schieffer B., Wirger A., Meybrunn M., Seitz S., Holtz J., Riede U.N., Drexler H. (1994). Comparative effects of chronic angiotensin-converting enzyme inhibition and angiotensin II type 1 receptor blockade on cardiac remodeling after myocardial infarction in the rat. Circulation.

[30] Aguilera G. (1992). Role of angiotensin II receptor subtypes on the regulation of aldosterone secretion in the adrenal glomerulosa zone in the rat. Mol. Cell. Endocrinol..

[31] Qadri F., Culman J., Veltmar A., Maas K., Rascher W., Unger T. (1993). Angiotensin II-induced vasopressin release is mediated through alpha-1 adrenoceptors and angiotensin II AT1 receptors in the supraoptic nucleus. J. Pharmacol. Exp. Ther..

[32] Sadoshima J.I., Izumo S. (1993). Molecular characterization of angiotensin II-induced hypertrophy of cardiac myocytes and hyperplasia of cardiac fibroblasts critical role of the AT1 receptor subtype. Circ. Res..

[33] Viswanathan M., Strömberg C., Seltzer A., Saavedra J.M. (1992). Balloon angioplasty enhances the expression of angiotensin II AT1 receptors in neointima of rat aorta. J. Clin. Investig..

[34] Jara Z.P., Icimoto M.Y., Yokota R., Ribeiro A.A., Dos Santos F., Souza L.E.D., Watanabe I.K.M., Franco M.D.C., Pesquero J.L., Irigoyen M.C. (2019). Tonin overexpression in mice diminishes sympathetic autonomic modulation and alters angiotensin type 1 receptor response. Front. Med..

[35] Kramár E.A., Krishnan R., Harding J.W., Wright J.W. (1998). Role of nitric oxide in angiotensin IV-induced increases in cerebral blood flow. Regul. Pept..

[36] Williams S., Malatesta K., Norris K. (2009). Vitamin D and Chronic Kidney Disease. Ethn. Dis..

[37] Freundlich M., Quiroz Y., Zhang Z., Zhang Y., Bravo Y., Weisinger J.R., Li Y.C., Rodriguez-Iturbe B. (2008). Suppression of renin–angiotensin gene expression in the kidney by paricalcitol. Kidney Int..

[38] Andress D.L. (2006). Vitamin D in chronic kidney disease: A systemic role for selective vitamin D receptor activation. Kidney Int..

[39] Tajalli-Nezhad S., Karimian M., Beyer C., Atlasi M.A., Tameh A.A. (2019). The regulatory role of Toll-like receptors after ischemic stroke: Neurosteroids as TLR modulators with the focus on TLR2/4. Cell. Mol. Life Sci..

[40] Wamberg L., Kampmann U., Stødkilde-Jørgensen H., Rejnmark L., Pedersen S.B., Richelsen B. (2013). Effects of vitamin D supplementation on body fat accumulation, inflammation, and metabolic risk factors in obese adults with low vitamin D levels-results from a randomized trial. Eur. J. Intern. Med..

[41] Li Y.C. (2010). Renoprotective effects of vitamin D analogs. Kidney Int..

[42] Hori M., Takahashi H., Kondo C., Hayashi F., Tokoroyama S., Mori Y., Tsujita M., Shirasawa Y., Takeda A., Morozumi K. (2024). Association between serum 25-hydroxyvitamin D levels and sarcopenia in patients undergoing chronic haemodialysis. Am. J. Nephrol..

[43] Wintermeyer E., Ihle C., Ehnert S., Stöckle U., Ochs G., De Zwart P., Flesch I., Bahrs C., Nussler A.K. (2016). Crucial Role of Vitamin D in the Musculoskeletal System. Nutrients.

[44] Simpson R.U., Thomas G.A., Arnold A.J. (1985). Identification of 1,25-dihydroxyvitamin D3 receptors and activities in muscle. J. Biol. Chem..

[45] Gallieni M.A.U.R.I.Z.I.O., Kamimura S.H.I.G.E.H.I.T.O., Ahmed A.D.N.A.N., Bravo E.R.I.C., Delmez J.A.M.E.S., Slatopolsky E.D.U.A.R.D.O., Dusso A.D.R.I.A.N.A. (1995). Kinetics of monocyte 1 alpha-hydroxylase in renal failure. Am. J. Physiol.-Ren. Physiol..

[46] Sabatino A., Cuppari L., Stenvinkel P., Lindholm B., Avesani C.M. (2021). Sarcopenia in chronic kidney disease: What have we learned so far?. J. Nephrol..

[47] Francis R., Aspray T., Fraser W., Sanjeev Patel M., Mavroeidi A., Schoenmakers I., Stone M. (2018). Vitamin D and Bone Health: A Practical Clinical Guideline for Patient Management. Natl. Osteoporos. Soc..

[48] Rosen C.J., Abrams S.A., Aloia J.F., Brannon P.M., Clinton S.K., Durazo-Arvizu R.A., Gallagher J.C., Gallo R.L., Jones G., Kovacs C.S. (2012). IOM Committee Members Respond to Endocrine Society Vitamin D Guideline. J. Clin. Endocrinol. Metab..

[49] Jones G. (2007). Expanding role for vitamin D in chronic kidney disease: Importance of blood 25-OH-D levels and extra-renal 1alpha-hydroxylase in the classical and nonclassical actions of 1alpha,25-dihydroxyvitamin D(3). Semin. Dial..

[50] Heaney R.P. (2008). Vitamin D in Health and Disease. Clin. J. Am. Soc. Nephrol..

[51] Holick M.F. (2009). Vitamin D status: Measurement, interpretation, and clinical application. Ann. Epidemiol..

[52] Filipov J.J., Zlatkov B.K., Dimitrov E.P., Svinarov D. (2015). Relationship between vitamin D status and immunosuppressive therapy in kidney transplant recipients. Biotechnol. Biotechnol. Equip..

[53] Shimada S., Hirose T., Takahashi C., Sato E., Kinugasa S., Ohsaki Y., Kisu K., Sato H., Ito S., Mori T. (2018). Pathophysiological and molecular mechanisms involved in renal congestion in a novel rat model. Sci. Rep..

[54] Faul C. (2018). FGF23 effects on the heart—Levels, time, source, and context matter. Kidney Int..

[55] Shimada T., Kakitani M., Yamazaki Y., Hasegawa H., Takeuchi Y., Fujita T., Fukumoto S., Tomizuka K., Yamashita T. (2004). Targeted ablation of Fgf23 demonstrates an essential physiological role of FGF23 in phosphate and vitamin D metabolism. J. Clin. Investig..

[56] Perwad F., Azam N., Zhang M.Y.H., Yamashita T., Tenenhouse H.S., Portale A.A. (2005). Dietary and Serum Phosphorus Regulate Fibroblast Growth Factor 23 Expression and 1,25-Dihydroxyvitamin D Metabolism in Mice. Endocrinology..

[57] Usatii M., Rousseau L., Demers C., Petit J.L., Brossard J.H., Gascon-Barré M., Lavigne J.R., Zahradnik R.J., Nemeth E.F., D’amour P. (2007). Parathyroid hormone fragments inhibit active hormone and hypocalcemia-induced 1,25(OH)2D synthesis. Kidney Int..

[58] Szymczak-Pajor I., Drzewoski J., Śliwińska A. (2020). The Molecular Mechanisms by Which Vitamin D Prevents Insulin Resistance and Associated Disorders. Int. J. Mol. Sci..

[59] Carey R.M., Siragy H.M. (2003). Newly Recognized Components of the Renin-Angiotensin System: Potential Roles in Cardiovascular and Renal Regulation. Endocr. Rev..

[60] Li P., He L., Sha Y., Luan Q. (2009). Relationship of Metabolic Syndrome to Chronic Periodontitis. J. Periodontol..

[61] Mehrotra R., Kermah D.A., Salusky I.B., Wolf M.S., Thadhani R.I., Chiu Y.W., Martins D., Adler S.G., Norris K.C. (2009). Chronic kidney disease, hypovitaminosis D, and mortality in the United States. Kidney Int..

[62] Kestenbaum B., Belozeroff V. (2007). Mineral metabolism disturbances in patients with chronic kidney disease. Eur. J. Clin. Investig..

[63] Ravani P., Malberti F., Tripepi G., Pecchini P., Cutrupi S., Pizzini P., Mallamaci F., Zoccali C. (2009). Vitamin D levels and patient outcome in chronic kidney disease. Kidney Int..

[64] Ho B.B., Bergwitz C. (2021). FGF23 signalling and physiology. J. Mol. Endocrinol..

[65] Wahl P., Wolf M. (2012). FGF23 in chronic kidney disease. Adv. Exp. Med. Biol..

[66] Jamal S.A., Miller P.D. (2013). Secondary and tertiary hyperparathyroidism. J. Clin. Densitom..

[67] Al-Aly Z., Qazi R.A., González E.A., Zeringue A., Martin K.J. (2007). Changes in serum 25-hydroxyvitamin D and plasma intact PTH levels following treatment with ergocalciferol in patients with CKD. Am. J. Kidney Dis..

[68] Rapa S.F., Di Iorio B.R., Campiglia P., Heidland A., Marzocco S. (2019). Inflammation and Oxidative Stress in Chronic Kidney Disease—Potential Therapeutic Role of Minerals, Vitamins and Plant-Derived Metabolites. Int. J. Mol. Sci..

[69] Nakai K., Fujii H., Kono K., Goto S., Kitazawa R., Kitazawa S., Hirata M., Shinohara M., Fukagawa M., Nishi S. (2014). Vitamin D Activates the Nrf2-Keap1 Antioxidant Pathway and Ameliorates Nephropathy in Diabetic Rats. Am. J. Hypertens..

[70] Lewis K.N., Mele J., Hayes J.D., Buffenstein R. (2010). Nrf2, a Guardian of Healthspan and Gatekeeper of Species Longevity. Integr. Comp. Biol..

[71] Tullet J.M., Green J.W., Au C., Benedetto A., Thompson M.A., Clark E., Gilliat A.F., Young A., Schmeisser K., Gems D. (2017). The SKN-1/Nrf2 transcription factor can protect against oxidative stress and increase lifespan in *C. elegans* by distinct mechanisms. Aging Cell.

[72] Razzaque M.S. (2012). FGF23, klotho and vitamin D interactions: What have we learned from in vivo mouse genetics studies?. Adv. Exp. Med. Biol..

[73] Lim K., Groen A., Molostvov G., Lu T., Lilley K.S., Snead D., James S., Wilkinson I.B., Ting S., Hsiao L.L. (2015). α-Klotho Expression in Human Tissues. J. Clin. Endocrinol. Metab..

[74] Fan Y., Cui C., Rosen C.J., Sato T., Xu R., Li P., Wei X., Bi R., Yuan Q., Zhou C. (2022). Klotho in Osx+-mesenchymal progenitors exerts pro-osteogenic and anti-inflammatory effects during mandibular alveolar bone formation and repair. Signal Transduct. Target. Ther..

[75] Zou D., Wu W., He Y., Ma S., Gao J. (2018). The role of klotho in chronic kidney disease. BMC Nephrol..

[76] Ni C., Bao D., Yan F., Chen B. (2023). Correlation between serum α-Klotho levels and different stages of periodontitis. BMC Oral Health.

[77] Zhao Y., Banerjee S., Dey N., LeJeune W.S., Sarkar P.S., Brobey R., Rosenblatt K.P., Tilton R.G., Choudhary S. (2011). Klotho Depletion Contributes to Increased Inflammation in Kidney of the *db/db* Mouse Model of Diabetes via RelA (Serine)536 Phosphorylation. Diabetes.

[78] Chen H., Huang X., Fu C., Wu X., Peng Y., Lin X., Wang Y. (2019). Recombinant Klotho Protects Human Periodontal Ligament Stem Cells by Regulating Mitochondrial Function and the Antioxidant System during H_2_O_2_ -Induced Oxidative Stress. Oxid. Med. Cell. Longev..

[79] França L.F.C., Vasconcelos A.C.C., da Silva F.R., Alves E.H., Carvalho J.S., Lenardo D.D., de Souza L.K., Barbosa A.L., Medeiros J.V.R., de Oliveira J.S. (2017). Periodontitis changes renal structures by oxidative stress and lipid peroxidation. J. Clin. Periodontol..

[80] Gyurászová M., Gurecká R., Bábíčková J., Tóthová L. (2020). Oxidative Stress in the Pathophysiology of Kidney Disease: Implications for Noninvasive Monitoring and Identification of Biomarkers. Oxid. Med. Cell. Longev..

[81] Yang X., Li Y., Li Y., Ren X., Zhang X., Hu D., Gao Y., Xing Y., Shang H. (2017). Oxidative Stress-Mediated Atherosclerosis: Mechanisms and Therapies. Front. Physiol..

[82] Hertiš Petek T., Petek T., Močnik M., Marčun Varda N. (2022). Systemic Inflammation, Oxidative Stress and Cardiovascular Health in Children and Adolescents: A Systematic Review. Antioxidants.

[83] Annuk M., Zilmer M., Lind L., Linde T., Fellström B. (2001). Oxidative Stress and Endothelial Function in Chronic Renal Failure. J. Am. Soc. Nephrol..

[84] Locatelli F., Canaud B., Eckardt K.U., Stenvinkel P., Wanner C., Zoccali C. (2003). Oxidative stress in end-stage renal disease: An emerging threat to patient outcome. Nephrol. Dial. Transplant..

[85] Berer A., Stöckl J., Majdic O., Wagner T., Kollars M., Lechner K., Geissler K., Oehler L. (2000). 1,25-Dihydroxyvitamin D3 inhibits dendritic cell differentiation and maturation in vitro. Exp. Hematol..

[86] Penna G., Adorini L. (2000). 1α,25-Dihydroxyvitamin D3 Inhibits Differentiation, Maturation, Activation, and Survival of Dendritic Cells Leading to Impaired Alloreactive T Cell Activation. J. Immunol..

[87] Ao T., Kikuta J., Ishii M. (2021). The effects of vitamin D on immune system and inflammatory diseases. Biomolecules.

[88] Gombart A.F., Borregaard N., Koeffler H.P. (2005). Human cathelicidin antimicrobial peptide (CAMP) gene is a direct target of the vitamin D receptor and is strongly up-regulated in myeloid cells by 1,25-dihydroxyvitamin D_3_. FASEB J..

[89] Yuk J.M., Shin D.M., Lee H.M., Yang C.S., Jin H.S., Kim K.K., Lee Z.W., Lee S.H., Kim J.M., Jo E.K. (2009). Vitamin D3 Induces Autophagy in Human Monocytes/Macrophages via Cathelicidin. Cell Host Microbe.

[90] Liu P.T., Stenger S., Li H., Wenzel L., Tan B.H., Krutzik S.R., Ochoa M.T., Schauber J., Wu K., Meinken C. (2006). Toll-like receptor triggering of a vitamin D-mediated human antimicrobial response. Science.

[91] Carlberg C. (2019). Vitamin D Signaling in the Context of Innate Immunity: Focus on Human Monocytes. Front. Immunol..

[92] Skrobot A., Demkow U., Wachowska M. (2018). Immunomodulatory Role of Vitamin D: A Review. Adv. Exp. Med. Biol..

[93] Ikeda U., Wakita D., Ohkuri T., Chamoto K., Kitamura H., Iwakura Y., Nishimura T. (2010). 1α,25-Dihydroxyvitamin D3 and all-trans retinoic acid synergistically inhibit the differentiation and expansion of Th17 cells. Immunol. Lett..

[94] Chen S., Sims G.P., Chen X.X., Gu Y.Y., Chen S., Lipsky P.E. (2007). Modulatory Effects of 1,25-Dihydroxyvitamin D3 on Human B Cell Differentiation. J. Immunol..

[95] Wang T.T., Nestel F.P., Bourdeau V., Nagai Y., Wang Q., Liao J., Tavera-Mendoza L., Lin R., Hanrahan J.W., Mader S. (2004). Cutting edge: 1,25-dihydroxyvitamin D3 is a direct inducer of antimicrobial peptide gene expression. J. Immunol..

[96] Diamond G., Beckloff N., Ryan L.K. (2008). Host defense peptides in the oral cavity and the lung: Similarities and differences. J. Dent. Res..

[97] Carlberg C. (2019). Vitamin D: A Micronutrient Regulating Genes. Curr. Pharm. Des..

[98] Bhan I., Powe C.E., Berg A.H., Ankers E., Wenger J.B., Karumanchi S.A., Thadhani R.I. (2012). Bioavailable vitamin D is more tightly linked to mineral metabolism than total vitamin D in incident hemodialysis patients. Kidney Int..

[99] Denburg M.R., Bhan I. (2015). Vitamin D-Binding Protein in Health and Chronic Kidney Disease. Semin. Dial..

[100] Dhaif Y.G., Garcia-Sanchez R., Albuquerque R., Lu E. (2023). The association between Vitamin D binding protein levels and periodontal status: A systematic review. J. Periodontal Res..

[101] Powe C.E., Evans M.K., Wenger J., Zonderman A.B., Berg A.H., Nalls M., Tamez H., Zhang D., Bhan I., Karumanchi S.A. (2013). Vitamin D–Binding Protein and Vitamin D Status of Black Americans and White Americans. N. Engl. J. Med..

[102] Robinson-Cohen C., Hoofnagle A.N., Ix J.H., Sachs M.C., Tracy R.P., Siscovick D.S., Kestenbaum B.R., de Boer I.H. (2013). Racial differences in the association of serum 25-hydroxyvitamin D concentration with coronary heart disease events. JAMA.

[103] Parikh A., Chase H.S., Vernocchi L., Stern L. (2014). Vitamin D resistance in chronic kidney disease (CKD). BMC Nephrol..

[104] Chun R.F., Lauridsen A.L., Suon L., Zella L.A., Pike J.W., Modlin R.L., Martineau A.R., Wilkinson R.J., Adams J., Hewison M. (2010). Vitamin D-binding protein directs monocyte responses to 25-hydroxy- and 1,25-dihydroxyvitamin D. J. Clin. Endocrinol. Metab..

[105] Taylor G.W., Sato M., Minagawa K., Yoshihara A., Iwasaki M., Ansai T. (2019). Effect of chronic kidney disease on progression of clinical attachment loss in older adults: A 4-year cohort study. J. Periodontol..

[106] Serni L., Caroti L., Barbato L., Nieri M., Serni S., Cirami C.L., Cairo F. (2023). Association between chronic kidney disease and periodontitis. A systematic review and metanalysis. Oral Dis..

[107] Cannata-Andía J.B., Martín-Carro B., Martín-Vírgala J., Rodríguez-Carrio J., Bande-Fernández J.J., Alonso-Montes C., Carrillo-López N. (2020). Chronic Kidney Disease-Mineral and Bone Disorders: Pathogenesis and Management. Calcif. Tissue Int..

[108] Costacurta M., Basilicata M., Marrone G., Di Lauro M., Campolattano V., Bollero P., Docimo R., Di Daniele N., Noce A. (2022). The Impact of Chronic Kidney Disease on Nutritional Status and Its Possible Relation with Oral Diseases. Nutrients.

[109] Allen M.R., Chen N.X., Ii V.H.G., Moe S.M. (2013). E-Mail Adverse Mandibular Bone Effects Associated with Kidney Disease Are Only Partially Corrected with Bisphosphonate and/or Calcium Treatment. Am. J. Nephrol..

[110] De Vries T.J., Huesa C. (2019). The Osteocyte as a Novel Key Player in Understanding Periodontitis Through its Expression of RANKL and Sclerostin: A Review. Curr. Osteoporos. Rep..

[111] Liu M., Kurimoto P., Zhang J., Niu Q.T., Stolina M., Dechow P.C., Feng J.Q., Hesterman J., Silva M.D., Ominsky M.S. (2018). Sclerostin and DKK1 Inhibition Preserves and Augments Alveolar Bone Volume and Architecture in Rats with Alveolar Bone Loss. J. Dent. Res..

[112] Li T.J., Wang R., Li Q.Y., Li C.Y., Jiang L. (2020). Sclerostin regulation: A promising therapy for periodontitis by modulating alveolar bone. Chin. Med. J..

[113] Asamiya Y., Tsuchiya K., Nitta K. (2016). Role of sclerostin in the pathogenesis of chronic kidney disease-mineral bone disorder. Ren. Replace. Ther..

[114] Fisher M.A., Taylor G.W., West B.T., McCarthy E.T. (2011). Bidirectional relationship between chronic kidney and periodontal disease: A study using structural equation modeling. Kidney Int..

[115] Lertpimonchai A., Rattanasiri S., Tamsailom S., Champaiboon C., Ingsathit A., Kitiyakara C., Limpianunchai A., Attia J., Sritara P., Thakkinstian A. (2019). Periodontitis as the risk factor of chronic kidney disease: Mediation analysis. J. Clin. Periodontol..

[116] Grubbs V., Vittinghoff E., Taylor G., Kritz-Silverstein D., Powe N., Bibbins-Domingo K., Ishani A., Cummings S.R. (2016). The association of periodontal disease with kidney function decline: A longitudinal retrospective analysis of the MrOS dental study. Nephrol. Dial. Transplant..

[117] Kajiwara K., Sawa Y., Fujita T., Tamaoki S. (2021). Immunohistochemical study for the expression of leukocyte adhesion molecules, and FGF23 and ACE2 in P. gingivalis LPS-induced diabetic nephropathy. BMC Nephrol..

[118] Nakano C., Hamano T., Fujii N., Matsui I., Tomida K., Mikami S., Inoue K., Obi Y., Okada N., Tsubakihara Y. (2012). Combined use of vitamin D status and FGF23 for risk stratification of renal outcome. Clin. J. Am. Soc. Nephrol..

[119] Wolf M. (2012). Update on fibroblast growth factor 23 in chronic kidney disease. Kidney Int..

[120] Ersin Kalkan R., Öngöz Dede F., Gökmenoğlu C., Kara C. (2018). Salivary fetuin-A, S100A12, and high-sensitivity C-reactive protein levels in periodontal diseases. Oral Dis..

[121] Jirak P., Stechemesser L., Moré E., Franzen M., Topf A., Mirna M., Paar V., Pistulli R., Kretzschmar D., Wernly B. (2019). Clinical implications of fetuin-A. Adv. Clin. Chem..

[122] Furugen R., Kawasaki K., Kitamura M., Maeda T., Saito T., Hayashida H. (2020). Association of low fetuin-A levels with periodontitis in community-dwelling adults. J. Oral Sci..

[123] Caglar K., Yilmaz M.I., Saglam M., Cakir E., Kilic S., Sonmez A., Eyileten T., Yenicesu M., Oguz Y., Tasar M. (2008). Serum Fetuin-A Concentration and Endothelial Dysfunction in Chronic Kidney Disease. Nephron Clin. Pract..

[124] Zhou Z., Ji Y., Ju H., Chen H., Sun M. (2019). Circulating Fetuin-A and Risk of All-Cause Mortality in Patients with Chronic Kidney Disease: A Systematic Review and Meta-Analysis. Front. Physiol..

[125] Bassey P.E., Numthavaj P., Rattanasiri S., Sritara P., McEvoy M., Ongphiphadhanakul B., Thakkinstian A. (2022). Causal association pathways between fetuin-A and kidney function: A mediation analysis. J. Int. Med. Res..

[126] Mahendra J., Palathingal P., Mahendra L., Alzahrani K.J., Banjer H.J., Alsharif K.F., Halawani I.F., Muralidharan J., Annamalai P.T., Verma S.S. (2022). Impact of Red Complex Bacteria and TNF-α Levels on the Diabetic and Renal Status of Chronic Kidney Disease Patients in the Presence and Absence of Periodontitis. Biology.

[127] Zhang J., Jiang H., Sun M., Chen J. (2017). Association between periodontal disease and mortality in people with CKD: A meta-analysis of cohort studies. BMC Nephrol..

[128] Tai Y.H., Chen J.T., Kuo H.C., Chang W.J., Wu M.Y., Dai Y.X., Liu W.C., Chen T.J., Wu H.L., Cherng Y.G. (2021). Periodontal disease and risk of mortality and kidney function decline in advanced chronic kidney disease: A nationwide population-based cohort study. Clin. Oral Investig..

[129] Delbove T., Gueyffier F., Juillard L., Kalbacher E., Maucort-Boulch D., Nony P., Grosgogeat B., Gritsch K. (2021). Effect of periodontal treatment on the glomerular filtration rate, reduction of inflammatory markers and mortality in patients with chronic kidney disease: A systematic review. PLoS ONE.

[130] da Silva T.A., Abreu L.G., Esteves Lima R.P. (2021). A meta-analysis on the effect of periodontal treatment on the glomerular filtration rate of chronic kidney disease individuals: A systematic review and meta-analysis was conducted to assess the impact of the periodontal treatment on the glomerular filtration rate of individuals with chronic kidney disease. Spec. Care Dentist..

[131] Almeida S., Figueredo C.M., Lemos C., Bregman R., Fischer R.G. (2017). Periodontal treatment in patients with chronic kidney disease: A pilot study. J. Periodontal Res..

[132] Jean G., Terrat J.C., Vanel T., Hurot J.M., Lorriaux C., Mayor B., Chazot C. (2008). Evidence for persistent vitamin D 1-alpha-hydroxylation in hemodialysis patients: Evolution of serum 1,25-dihydroxycholecalciferol after 6 months of 25-hydroxycholecalciferol treatment. Nephron Clin. Pract..

[133] Walker J.P., Hiramoto J.S., Gasper W.J., Auyang P., Conte M.S., Rapp J.H., Lovett D.H., Owens C.D. (2014). Vitamin D deficiency is associated with mortality and adverse vascular access outcomes in patients with end-stage renal disease. J. Vasc. Surg..

[134] Lopez A.G., Kerlan V., Desailloud R. (2021). Non-classical effects of vitamin D: Non-bone effects of vitamin D. Ann. Endocrinol..

[135] Melamed M.L., Astor B., Michos E.D., Hostetter T.H., Powe N.R., Muntner P. (2009). 25-Hydroxyvitamin D levels, race, and the progression of kidney disease. J. Am. Soc. Nephrol..

[136] Pilz S., Tomaschitz A., März W., Drechsler C., Ritz E., Zittermann A., Cavalier E., Pieber T.R., Lappe J.M., Grant W.B. (2011). Vitamin D, cardiovascular disease and mortality. Clin. Endocrinol..

[137] Jayedi A., Soltani S., Shab-Bidar S. (2017). Vitamin D status and all-cause mortality in patients with chronic kidney disease: A systematic review and dose-response meta-analysis. J. Clin. Endocrinol. Metab..

[138] DeVille J., Thorp M.L., Tobin L., Gray E., Johnson E.S., Smith D.H. (2006). Effect of ergocalciferol supplementation on serum parathyroid hormone and serum 25-hydroxyvitamin D in chronic kidney disease. Nephrology.

[139] Ikizler T.A., Cuppari L. (2021). The 2020 Updated KDOQI Clinical Practice Guidelines for Nutrition in Chronic Kidney Disease. Blood Purif..

[140] Chowdhury R., Kunutsor S., Vitezova A., Oliver-Williams C., Chowdhury S., Kiefte-de-Jong J.C., Khan H., Baena C.P., Prabhakaran D., Hoshen M.B. (2014). Vitamin D and risk of cause specific death: Systematic review and meta-analysis of observational cohort and randomised intervention studies. BMJ.

[141] Duranton F., Rodriguez-Ortiz M.E., Duny Y., Rodriguez M., Daurès J.P., Argilés A. (2013). Vitamin D treatment and mortality in chronic kidney disease: A systematic review and meta-analysis. Am. J. Nephrol..

[142] Cheng S., Coyne D. (2007). Vitamin D and outcomes in chronic kidney disease. Curr. Opin. Nephrol. Hypertens..

[143] Armas L.A.G., Hollis B.W., Heaney R.P. (2004). Vitamin D_2_ Is Much Less Effective than Vitamin D_3_ in Humans. J. Clin. Endocrinol. Metab..

[144] Christodoulou M., Aspray T.J., Schoenmakers I. (2021). Vitamin D Supplementation for Patients with Chronic Kidney Disease: A Systematic Review and Meta-analyses of Trials Investigating the Response to Supplementation and an Overview of Guidelines. Calcif. Tissue Int..

[145] Dietrich T., Joshipura K.J., Dawson-Hughes B., Bischoff-Ferrari H.A. (2004). Association between serum concentrations of 25-hydroxyvitamin D_3_ and periodontal disease in the US population. Am. J. Clin. Nutr..

[146] Krall E.A., Wehler C., Garcia R.I., Harris S.S., Dawson-Hughes B. (2001). Calcium and vitamin D supplements reduce tooth loss in the elderly. Am. J. Med..

[147] Gao W., Tang H., Wang D., Zhou X., Song Y., Wang Z. (2020). Effect of short-term vitamin D supplementation after nonsurgical periodontal treatment: A randomized, double-masked, placebo-controlled clinical trial. J. Periodontal Res..

[148] Mishra S.M., Ravishankar P.L., Pramod V., Rajula P.B., Gayathri K., Alam M.K., Raj A.T., Bhandi S., Patil S. (2022). Effect of Supplementation of Vitamin D in Patients with Periodontitis Evaluated before and after Nonsurgical Therapy. Biomed. Res. Int..

[149] Hiremath V., Rao C., Naiak V., Prasad K.V.V. (2013). Anti-inflammatory effect of vitamin D on gingivitis: A dose response randomised controlled trial. Indian J. Public Health.

[150] Perić M., Maiter D., Cavalier E., Lasserre J.F., Toma S. (2020). The effects of 6 month Vitamin D supplementation during the non-surgical treatment of periodontitis in Vitamin D–deficient patients: A randomised double-blind placebo-controlled study. Nutrients.

[151] Bashutski J.D., Eber R.M., Kinney J.S., Benavides E., Maitra S., Braun T.M., Giannobile W.V., McCauley L.K. (2010). Teriparatide and Osseous Regeneration in the Oral Cavity. N. Engl. J. Med..

[152] Bashutski J.D., Eber R.M., Kinney J.S., Benavides E., Maitra S., Braun T.M., Giannobile W.V., McCauley L.K. (2011). The Impact of Vitamin D Status on Periodontal Surgery Outcomes. J. Dent. Res..

[153] Machado V., Lobo S., Proença L., Mendes J.J., Botelho J. (2020). Vitamin D and Periodontitis: A Systematic Review and Meta-Analysis. Nutrients.

[154] Bastos J.D.A., Andrade L.C.F.D., Ferreira A.P., Barroso E.D.A., Daibert P.D.C., Barreto P.L.D.S., Vilela E.M., Marcaccini A.M., Colugnati F.A.B., Bastos M.G. (2013). Serum levels of vitamin D and chronic periodontitis in patients with chronic kidney disease. J. Bras. Nefrol..

[155] Bostanci N. (2023). Periodontal health and pregnancy outcomes: Time to deliver. Acta Obstet. Gynecol. Scand..

[156] Ercan E., Eratalay K., Deren O., Gur D., Ozyuncu O., Altun B., Kanlı C., Ozdemir P., Akıncıbay H. (2013). Evaluation of periodontal pathogens in amniotic fluid and the role of periodontal disease in pre-term birth and low birth weight. Acta Odontol. Scand..

[157] Raju K., Berens L. (2021). Periodontology and pregnancy: An overview of biomedical and epidemiological evidence. Periodontol. 2000.

[158] Bobetsis Y.A., Graziani F., Gürsoy M., Madianos P.N. (2020). Periodontal disease and adverse pregnancy outcomes. Periodontol. 2000.

[159] Hebisch G., Grauaug A.A., Neumaier-Wagner P.M., Stallmach T., Huch A., Huch R. (2001). The relationship between cervical dilatation, interleukin-6 and interleukin-8 during term labor. Acta Obstet. Gynecol. Scand..

[160] Ferrillo M., Migliario M., Roccuzzo A., Molinero-Mourelle P., Falcicchio G., Umano G.R., Pezzotti F., Foglio Bonda P.L., Calafiore D., de Sire A. (2021). Periodontal Disease and Vitamin D Deficiency in Pregnant Women: Which Correlation with Preterm and Low-Weight Birth?. J. Clin. Med..

[161] Wang S., Xin X., Luo W., Mo M., Si S., Shao B., Shen Y., Cheng H., Yu Y. (2021). Association of vitamin D and gene variants in the vitamin D metabolic pathway with preterm birth. Nutrition.

[162] Lian R.H., Qi P.A., Yuan T., Yan P.J., Qiu W.W., Wei Y., Hu Y.G., Yang K.H., Yi B. (2021). Systematic review and meta-analysis of vitamin D deficiency in different pregnancy on preterm birth: Deficiency in middle pregnancy might be at risk. Medicine.

[163] MacRae C., Mercer S.W., Guthrie B., Henderson D. (2021). Comorbidity in chronic kidney disease: A large cross-sectional study of prevalence in Scottish primary care. Br. J. Gen. Pract..

[164] Dietrich T., Sharma P., Walter C., Weston P., Beck J. (2013). The epidemiological evidence behind the association between periodontitis and incident atherosclerotic cardiovascular disease. J. Clin. Periodontol..

[165] Hajishengallis G. (2022). Interconnection of periodontal disease and comorbidities: Evidence, mechanisms, and implications. Periodontol. 2000.

[166] Wang T.J., Pencina M.J., Booth S.L., Jacques P.F., Ingelsson E., Lanier K., Benjamin E.J., D’Agostino R.B., Wolf M., Vasan R.S. (2008). Vitamin D deficiency and risk of cardiovascular disease. Circulation.

[167] Zittermann A., Trummer C., Theiler-schwetz V., Lerchbaum E., März W., Pilz S. (2021). Vitamin d and cardiovascular disease: An updated narrative review. Int. J. Mol. Sci..

[168] Chen S., Glenn D.J., Ni W., Grigsby C.L., Olsen K., Nishimoto M., Law C.S., Gardner D.G. (2008). Expression of the vitamin D receptor is increased in the hypertrophic heart. Hypertension.

[169] Zittermann A., Schulze Schleithoff S., Tenderich G., Berthold H.K., Körfer R., Stehle P. (2003). Low vitamin D status: A contributing factor in the pathogenesis of congestive heart failure?. J. Am. Coll. Cardiol..

[170] Somjen D., Weisman Y., Kohen F., Gayer B., Limor R., Sharon O., Jaccard N., Knoll E., Stern N. (2005). 25-Hydroxyvitamin D3-1α-hydroxylase is expressed in human vascular smooth muscle cells and is upregulated by parathyroid hormone and estrogenic compounds. Circulation.

[171] Friedlaender M.M., Kornberg Z., Wald H., Popovtzer M.M. (1983). Renal effect of vitamin D metabolites: Evidence for the essential role of the 25(OH) group. Am. J. Physiol.-Ren. Physiol..

[172] Elidrissy A.T.H., Munawarah M., Alharbi K.M. (2013). Hypocalcemic rachitic cardiomyopathy in infants. J. Saudi Heart Assoc..

[173] Subbiah A.K., Chhabra Y.K., Mahajan S. (2016). Cardiovascular disease in patients with chronic kidney disease: A neglected subgroup. Heart Asia.

[174] Nelson A.J., Raggi P., Wolf M., Gold A.M., Chertow G.M., Roe M.T. (2020). Targeting Vascular Calcification in Chronic Kidney Disease. JACC Basic Transl. Sci..

[175] Kolb H., Mandrup-Poulsen T. (2005). An immune origin of type 2 diabetes?. Diabetologia.

[176] Tuttle K.R., Bakris G.L., Bilous R.W., Chiang J.L., De Boer I.H., Goldstein-Fuchs J., Hirsch I.B., Kalantar-Zadeh K., Narva A.S., Navaneethan S.D. (2014). Diabetic kidney disease: A report from an ADA consensus conference. Diabetes Care.

[177] Fernandez-Juarez G., Luno J., Barrio V., de Vinuesa S.G., Praga M., Goicoechea M., Lahera V., Casas L., Oliva J. (2013). 25 (OH) vitamin D levels and renal disease progression in patients with type 2 diabetic nephropathy and blockade of the renin-angiotensin system. Clin. J. Am. Soc. Nephrol..

[178] Shultis W.A., Weil E.J., Looker H.C., Curtis J.M., Shlossman M., Genco R.J., Knowler W.C., Nelson R.G. (2007). Effect of periodontitis on overt nephropathy and end-stage renal disease in type 2 diabetes. Diabetes Care.

[179] Naruishi K., Oishi K., Inagaki Y., Horibe M., Bando M., Ninomiya M., Kawahara K., Minakuchi J., Kawashima S., Shima K. (2016). Association between periodontal condition and kidney dysfunction in Japanese adults: A cross-sectional study. Clin. Exp. Dent. Res..

[180] Andrukhov O., Andrukhova O., Hulan U., Tang Y., Bantleon H.P., Rausch-Fan X. (2014). Both 25-hydroxyvitamin-D_3_ and 1,25-dihydroxyvitamin- D_3_ reduces inflammatory response in human periodontal ligament cells. PLoS ONE.

[181] Lips P., Eekhoff M., van Schoor N., Oosterwerff M., de Jongh R., Krul-Poel Y., Simsek S. (2017). Vitamin D and type 2 diabetes. J. Steroid Biochem. Mol. Biol..

[182] Muñoz-Garach A., García-Fontana B., Muñoz-Torres M. (2019). Vitamin D status, calcium intake and risk of developing type 2 diabetes: An unresolved issue. Nutrients.

[183] Berridge M.J. (2017). Vitamin D deficiency and diabetes. Biochem. J..

[184] Berridge M.J. (2016). Vitamin D, reactive oxygen species and calcium signalling in ageing and disease. Philos. Trans. R. Soc. B Biol. Sci..

[185] Jain S.K., Micinski D. (2013). Vitamin D upregulates glutamate cysteine ligase and glutathione reductase, and GSH formation, and decreases ROS and MCP-1 and IL-8 secretion in high-glucose exposed U937 monocytes. Biochem. Biophys. Res. Commun..

[186] Tagliaferri S., Porri D., De Giuseppe R., Manuelli M., Alessio F., Cena H. (2019). The controversial role of Vitamin D as an antioxidant: Results from randomised controlled trials. Nutr. Res. Rev..

[187] Park S., Kim D.S., Kang S. (2016). Vitamin D deficiency impairs glucose-stimulated insulin secretion and increases insulin resistance by reducing PPAR-γ expression in nonobese Type 2 diabetic rats. J. Nutr. Biochem..

[188] Takiishi T., Gysemans C., Bouillon R., Mathieu C. (2010). Vitamin D and diabetes. Endocrinol. Metab. Clin. N. Am..

[189] Kang S., Tsai L.T., Zhou Y., Evertts A., Xu S., Griffin M.J., Issner R., Whitton H.J., Garcia B.A., Epstein C.B. (2015). Identification of nuclear hormone receptor pathways causing insulin resistance by transcriptional and epigenomic analysis. Nat. Cell Biol..

[190] Ong L.T.C., Booth D.R., Parnell G.P. (2020). Vitamin D and its Effects on DNA Methylation in Development, Aging, and Disease. Mol. Nutr. Food Res..

[191] Murdaca G., Tonacci A., Negrini S., Greco M., Borro M., Puppo F., Gangemi S. (2019). Emerging role of vitamin D in autoimmune diseases: An update on evidence and therapeutic implications. Autoimmun. Rev..

[192] Mouterde G., Gamon E., Rincheval N., Lukas C., Seror R., Berenbaum F., Dupuy A.M., Daien C., Daurès J.P., Combe B. (2020). Association Between Vitamin D Deficiency and Disease Activity, Disability, and Radiographic Progression in Early Rheumatoid Arthritis: The ESPOIR Cohort. J. Rheumatol..

[193] Rossini M., Maddali Bongi S., La Montagna G., Minisola G., Malavolta N., Bernini L., Cacace E., Sinigaglia L., Di Munno O., Adami S. (2010). Vitamin D deficiency in rheumatoid arthritis: Prevalence, determinants and associations with disease activity and disability. Arthritis Res. Ther..

[194] Caraba A., Crişan V., Romoşan I., Mozoş I., Murariu M. (2017). Vitamin D status, disease activity, and endothelial dysfunction in early rheumatoid arthritis patients. Dis. Markers.

[195] Dankers W., Davelaar N., Asmawidjaja P.S., Mus A.M., Hazes J.M., Colin E.M., Lubberts E. THU0047 1,25(OH)2d3 and dexamethasone additively suppress synovial fibroblast activation by ccr6+ th memory cells and enhance the effect of tnf-alpha blockade. Proceedings of the Annual European Congress of Rheumatology, EULAR 2018.

[196] Mok C.C. (2018). Systemic lupus erythematosus: What should family physicians know in 2018?. Hong Kong Med. J..

[197] Schoindre Y., Jallouli M., Tanguy M.L., Ghillani P., Galicier L., Aumaître O., Francès C., Le Guern V., Lioté F., Smail A. (2014). Lower Vitamin D levels are associated with higher systemic lupus erythematosus activity, but not predictive of disease flare-up. Lupus Sci. Med..

[198] Linker-Israeli M., Elstner E., Klinenberg J.R., Wallace D.J., Koeffler H.P. (2001). Vitamin D3 and its synthetic analogs inhibit the spontaneous in vitro immunoglobulin production by SLE-derived PBMC. Clin. Immunol..

[199] Sellner J., Kraus J., Awad A., Milo R., Hemmer B., Stüve O. (2011). The increasing incidence and prevalence of female multiple sclerosis-A critical analysis of potential environmental factors. Autoimmun. Rev..

[200] Hiremath G.S., Cettomai D., Baynes M., Ratchford J.N., Newsome S., Harrison D., Kerr D., Greenberg B.M., Calabresi P.A. (2009). Vitamin D status and effect of low-dose cholecalciferol and high-dose ergocalciferol supplementation in multiple sclerosis. Mult. Scler..

[201] Lemire J.M., Ince A., Takashima M. (1992). 1,25-dihydroxyvitamin d3 attenuates of expression of experimental murine lupus of MRL/1 mice. Autoimmunity.

[202] Mattner F., Smiroldo S., Galbiati F., Muller M., Di Lucia P., Poliani P.L., Martino G., Panina-Bordignon P., Adorini L. (2000). Inhibition of Th1 development and treatment of chronic-relapsing experimental allergic encephalomyelitis by a non-hypercalcemic analogue of 1,25-dihydroxyvitamin D3. Eur. J. Immunol..

[203] Fichna M., Żurawek M., Januszkiewicz-Lewandowska D., Gryczynska M., Fichna P., Sowinski J., Nowak J. (2010). Association of the CYP27B1 C(-1260)A polymorphism with autoimmune Addison’s disease. Exp. Clin. Endocrinol. Diabetes.

[204] Yazici D., Yavuz D., Tarcin O., Sancak S., Deyneli O., Akalin S. (2013). Vitamin D receptor gene ApaI, TaqI, FokI and BsmI polymorphisms in a group of Turkish patients with Hashimoto’s thyroiditis. Minerva Endocrinol..

[205] Zhang J., Li W., Liu J., Wu W., Ouyang H., Zhang Q., Wang Y., Liu L., Yang R., Liu X. (2012). Polymorphisms in the vitamin D receptor gene and type 1 diabetes mellitus risk: An update by meta-analysis. Mol. Cell. Endocrinol..

